# Clinical study of the influence of preoperative wearing time on postoperative effects in children with partially accommodative esotropia

**DOI:** 10.1097/MD.0000000000010619

**Published:** 2018-05-11

**Authors:** Danni Jiang, Dong Han, Jiahuan Zhang, Tianxu Pei, Qi Zhao

**Affiliations:** The Second Hospital of Dalian Medical University, Dalian, Liaoning, China.

**Keywords:** children, partially accommodative esotropia, postoperative effect, preoperative wearing time

## Abstract

The aim of this study was to evaluate the influence of the preoperative wearing time on the postoperative effect in children with partially accommodative esotropia.

Sixty children with partially accommodative esotropia who visited our hospital were placed in full cycloplegic refraction by using 1% Atropine eye gel and then wore full hyperopic correction glasses. Children were divided into groups A and B according to the preoperative wearing time. The visual acuity, eye position, and results of the synoptophore and Titmus stereoacuity tests were recorded before and half a year after the surgery in each group, and appropriate statistical analyses were conducted.

Half a year after the operation, 54 cases achieved orthotropia when wearing full hyperopic correction glasses. One case was overcorrected. Five cases were undercorrected. The results of the synoptophore and Titmus stereoacuity test showed that there was no significant difference between postoperative outcomes for patients who wore glasses for half a year and for 1 year before the operation.

For children with partially accommodative esotropia, surgery should be used to correct the eye position after wearing full hyperopic correction glasses for half a year to improve the eye position and binocular vision as early as possible. If the operation cannot be completed after the patient wears full hyperopic correction glasses for half a year due to various subjective and objective factors, a good postoperative effect can be obtained if the patients receive surgery after wearing full hyperopic correction glasses for 1 year.

## 1 Introduction

Partially accommodative esotropia is a common type of concomitant esotropia in the clinic that occurs in approximately one-third of concomitant esotropia cases.^[1]^ The cause of the disease includes accommodative factors and nonaccommodative factors.^[2]^ The accommodative factors are often caused by different degrees of hypermetropia.^[3]^ The nonaccommodative factors include anatomy factors, congenital fusion dysfunction, or anatomical contracture of the medial rectus muscle caused by the absence of an effective treatment in fully accommodative esotropia.^[4]^ The deviation angle caused by the accommodative part needs to be corrected by wearing full hyperopic correction glasses. For children with amblyopia, amblyopia should be treated at the same time. Clinical studies have shown that deviation angles in children obviously decrease after wearing full hyperopic correction glasses for a period of time, but the residual esotropia requires surgical correction. Because strabismus seriously affects the development of binocular vision in children, performing the operation too late can lead to difficultly in children's reconstructing binocular vision.^[5]^ Therefore, we cannot wait long time for the cure of amblyopia to operate.^[6]^ However, performing the operation too early causes the nonaccommodative part of the deviation angle to become unstable and increases the possibility of exotropia after the operation.^[7]^ Therefore, how long should patients wear full hyperopic correction glasses before the operation? Some scholars believe that the residual part that cannot be corrected by wearing full hyperopic correction glasses should be surgically corrected as soon as possible and that operating soon is very important for the recovery of binocular vision.^[8]^ Other scholars believe that children with partially accommodative esotropia should be fully observed and we should treat the nonaccommodative part after the deviation angle is stabilized.^[9]^ To analyze the influence of the preoperative wearing time on the postoperative outcomes, we divided children with partially accommodative esotropia who visited our hospital into 2 groups according to the preoperative wearing time. One group included children who wore full hyperopic correction glasses for half a year before the operation, and the other group included children who wore full hyperopic correction glasses for 1 year before the operation. The postoperative effects of the 2 groups were compared and analyzed to find the appropriate time for partially accommodative esotropia to be surgically corrected in children.

## 2 Materials and methods

### 2.1 Materials

Sixty children with partially accommodative esotropia who visited our hospital between October 2015 and October 2016 were eligible for the study. The study population included 32 female and 28 male patients. The mean age was 6.2 years (range, 3–11 years). The inclusion criteria were children whose near or far strabism angle decreased after wearing full hyperopic correction glasses for half a year or 1 year, but had a condition that could not be completely eliminated and a residual strabismus angle that was more than +10△. Children were divided into groups A (30 cases) and B (30 cases) according to their preoperative wearing time. Children in group A wore glasses for half a year before surgery and had deviation angles that were stable. Children in group B wore glasses for 1 year before surgery and had deviation angles that were stable.

### 2.2 Methods

The work was carried out in accordance with The Code of Ethics of the World Medical Association (Declaration of Helsinki). Informed consent was obtained from the parents of the children and privacy rights was fully respected. First of all, anterior chamber and fundus examinations were performed for all patients, and other eye diseases were excluded. Then, we used 1% Atropine eye gel for 3 days to produce mydriasis. All patients had either hyperopia or hyperopia with astigmatism, and 18 cases had amblyopia. All patients wore full hyperopic correction glasses according to the result of the mydriasis optometry, and amblyopia was treated actively. We tested the best corrected visual acuity according to the International standard visual acuity chart before the operation. And the spherical equivalent was from +1.00DS to + 7.00DS.

#### 2.2.1 Eye position

A simultaneous prism cover test was used to measure the strabismus angle of children at 33 cm fixation and 5 m fixation and the test for ocular alignment were performed with and without full hyperopic correction glasses. We used the synoptophore to evaluate the 3-level visual function of far stereopsis and measurement of near stereopsis was recorded by using the Titmus fly test before surgery.

The design of the operation was carried out according to the residual strabismus along with full hyperopic correction glasses. Patients with an esotropia angle of + 20^△^ or less were treated with unilateral medial rectus recession. Patients with an esotropia angle that was from + 25^△^ to + 45^△^ were treated with bilateral medial rectus recession. Patients with an esotropia angle that was more than + 50^△^ were treated with bilateral medial rectus recession and unilateral external rectus shortening.^[10]^ In this study, 4 cases of nondominant medial rectus recession were performed, 23 cases of bilateral medial rectus recession were performed, and 3 cases of bilateral medial rectus recession and nondominant external rectus shortening were performed.

All surgeries were performed by professor Qi Zhao under general anesthesia. The operation was begun through conjunctival fornix incisions. During the operation of medial rectus recession, first, we separated the fascia and check ligaments, then sutured the medial rectus muscle by a double loop at 1 mm away from the muscle insertion, cut the medial rectus muscle near the insertion, and recessed 3∼5 mm; finally, we closed the conjunctiva. For children who needed bilateral medial rectus recession to correct the eye position, we did the same treatment in the other eye. The sutures we used were double-armed 6–0 polyglactin 910 (Vicryl, Ethicon, Somerville, NJ) sutures with spatula needle. For patients with esotropia angle that was more than + 50△, in addition to the above procedures, in nondominant eye, we needed to suture the external rectus muscle by a double loop at 2∼8 mm away from the muscle insertion and cut the muscle next to the double loop, resected the muscle, and fixed on the insertion. Finally, we closed the conjunctiva.

Follow up was conducted with all patients for no less than half a year, the glasses were adjusted according to the postoperative eye position to maintain the eye position normotopia. At the same time, the corrected vision, eye position, synoptophore and Titmus stereoacuity test results were recorded. The postoperative squint angle with glasses between -8^△^ and + 8^△^ was defined orthophoria. The postoperative squint angle with glasses more than -8^△^ was defined overcorrection. The postoperative squint angle with glasses more than + 8^△^ was defined undercorrection.^[11]^

### 2.3 Statistical method

We used SPSS software (ver. 22.0; IBM Corp, Armonk, NY) to analyze the data. Independent-samples *t* test was adopted to compare the preoperative corrected visual acuity between A and B group, the postoperative corrected visual acuity between A and B group, and the corrected visual acuity before and after surgery. Chi-square test was adopted to compare the postoperative eye position between the 2 groups, the result of synoptophore and Titmus stereoacuity test before and after surgery, and so on. *P* < .05 was considered statistically significant.

## 3 Results

In all our cases, before the operation, the best corrected visual acuity in 25 eyes was no less than 0.9, and there were 13 eyes from group A and 12 eyes from group B. The best corrected visual acuity in 68 eyes ranged from 0.6 to 0.8, and there were 33 eyes from group A and 35 eyes from group B. Twenty-seven eyes had a best corrected visual acuity that was no more than 0.5, and there were 14 eyes from group A and 13 eyes from group B. There was no statistically significant difference between the corrected visual acuity of 2 groups before surgery (*P* = .60). Half a year after the operation, 28 eyes had a best corrected visual acuity that was no less than 0.9, and there were 14 eyes from group A and 14 eyes from group B. The best corrected visual acuity in 66 eyes ranged from 0.6 to 0.8, and there were 32 eyes from group A and 34 eyes from group B. The best corrected visual acuity in 26 eyes was no more than 0.5, and there were 14 eyes in group A and 12 eyes in group B. There was no statistically significant difference between the 2 groups half a year after surgery as well (*P* = .77). The results showed that there was no significant difference in the preoperative or postoperative corrected visual acuity between the children who wore glasses for half a year and for 1 year before surgery. Comparing the best corrected visual acuity before and half a year after surgery, there was no significant difference (*P* = .17).

In all our cases, before the operation, near angle without correction: + 25△—+ 120△, average+ 53.25△; near angle with correction: + 15△— + 80△, average + 42.63△; distant angle without correction: + 20△— + 120△, average + 52.14△; distant angle with correction: + 15△— + 80△, average + 40.63△. The average difference in the near angle between with and without full hyperopic correction glasses was 10.62△, and the average difference in the distant angle between with and without full hyperopic correction glasses was 11.51△. Half a year after the operation, the eye position of 54 cases (90%) was orthophoria. There were 26 cases (86.67%) in group A and 28 cases (93.33%) in group B. There was 1 case with overcorrection, and the overcorrection incidence was 1.67%. This case was from group B (3.33%). There were 5 cases of undercorrection, and the undercorrection incidence was 8.33%. There were 4 cases (13.33%) from group A and 1 case (3.33%) from group B (see Table 1 for the comparison of postoperative eye position). There was no statistically significant difference between the 2 groups (*P* = .57). The results showed that there was no significant difference in the postoperative eye position between the children who wore glasses for half a year and for 1 year before surgery.

**Table 1:**
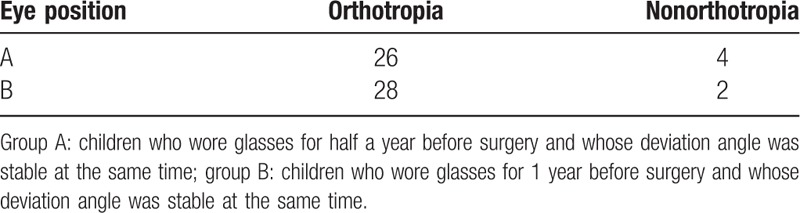
The comparison of postoperative eye position between group A and group B.

3.1 The preoperative results of the synoptophore test

For group A, 18 cases (60%) had no simultaneous view, 9 cases (30%) had primary visual function, 2 cases (6.67%) had fusion function and 1 case (3.33%) had stereopsis function. For group B, 18 cases (60%) had no simultaneous view, 7 cases (23.33%) had primary visual function, 3 cases (10%) had fusion function, and 2 cases (6.67%) had stereopsis function. There was no statistically significant difference between the 2 groups of synoptophore's result before surgery (*P* = .68). Half a year after the operation, the synoptophore results showed that 15 cases (25%) had no binocular visual function, 5 cases (8.33%) had primary visual function, 8 cases (13.33%) had fusion function, and 32 cases (53.33%) had stereopsis function. There were 45 cases (75%) that had binocular visual function in total (see Table 2 for the comparison of preoperative and postoperative binocular visual function). Compared with the preoperative results, postoperative binocular visual function increased significantly, and the difference was statistically significant (*P* < .001). For group A, 10 cases (33.33%) had no binocular visual function, 2 cases (6.67%) had primary visual function, 4 cases (13.33%) had fusion function, and 14 cases (46.67%) had stereopsis function. For group B, 5 cases (16.67%) had no binocular visual function, 3 cases (10%) had primary visual function, 4 cases (13.33%) had fusion function, and 18 cases (60%) had stereopsis function (see Table 3 for the comparison of 2 groups’ postoperative binocular visual function). There was no statistically significant difference between the two groups (*P* = .38). The results of this study showed that there was no significant difference in postoperative binocular visual function between children who wore glasses for half a year and for 1 year before surgery.

**Table 2:**
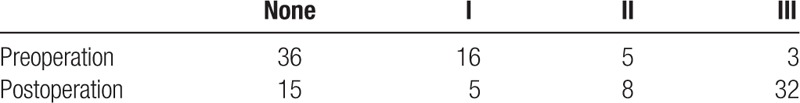
The comparison of synoptophore's result between preoperation and postoperation.

**Table 3:**
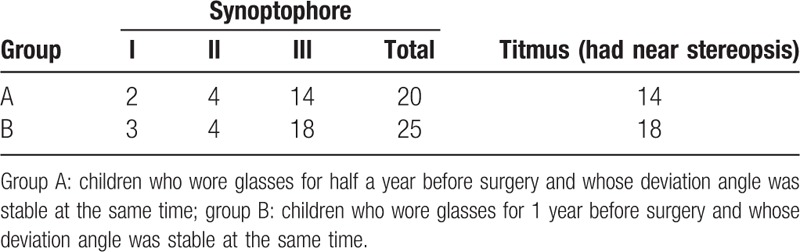
The comparison of postoperative binocular visual function between group A and group B.

3.2 The preoperative results of the Titmus stereoacuity test

For group A, 3 cases (10%) had the near stereopsis function. For group B, 3 cases (10%) had the near stereopsis function. There was no statistically significant difference between the 2 groups of Titmus stereoacuity test's result before surgery (*P* = .54). Half a year after surgery, the results showed that 28 cases (46.67%) did not have near stereopsis function and 32 cases (53.33%) had near stereopsis function (see Table 4 for the comparison of preoperative and postoperative near stereopsis function). Compared with the preoperative results, the postoperative near stereopsis function increased significantly as well, and the difference was statistically significant (*P* = .02). For group A, 14 cases (46.67%) had near stereopsis function, and for group B, 18 cases (60%) had near stereopsis function (see Table 3 for the comparison of 2 groups’ postoperative near stereopsis function). There was no statistically significant difference between the 2 groups (*P* = .30). The results showed that the postoperative near stereopsis function had no significant difference between children who wore glasses for half a year and for 1 year before surgery.

**Table 4:**
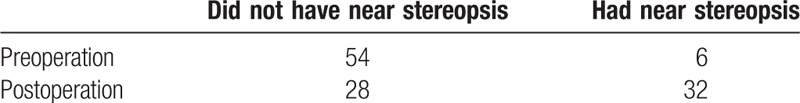
The comparison of Titmus stereoacuity test's result between preoperation and postoperation.

## 4 Discussion

Partially accommodative esotropia is a common type of concomitant esotropia that usually occurs in children under 12 years of age. The age of onset is usually from 1 to 3 years.^[12]^ The causes of the disease are complicated and often include both accommodative and nonaccommodative factors. The accommodative factors are often caused by uncorrected hyperopia. The nonaccommodative factors include anatomic factors, congenital fusion dysfunction, and internal rectus muscle dysfunction due to the absence of an effective treatment in fully accommodative esotropia. For the deviation angle caused by the accommodative factor, we used 1% atropine eye gel for 3 days and achieved cycloplegia. Then, we required children to wear full hyperopic correction glasses to correct their eye position. Wearing full hyperopic correction glasses not only promotes improvement of distance vision but also maximumly removes the accommodative component to decrease the influence of strabismus on binocular vision.^[13]^ After wearing the glasses for a designated period of time, the nonaccommodative component that cannot be corrected by the lens requires surgical correction.

Partially accommodative esotropia is not only accompanied by hyperopia but also by amblyopia. Children with amblyopia require active treatment while wearing full hyperopic correction glasses. Several studies have shown that surgical treatment should be performed as soon as the disparity of binocular visual acuity is less than 2 lines and the esotropia angle with glasses is stable. We also need to actively treat amblyopia after the surgery.^[14]^ Surgery should be performed without waiting for correction of amblyopia, if we wait for the amblyopia to be resolved for too long time, the best time for the operation will be missed and reconstruction of binocular visual function will be delayed.

Binocular visual function is an advanced visual function that is formed through environmental cognition. It is the process of using both eyes synchronously to recognize an image that falls on the macular region of the retina and integrating the visual information from the eyes to form a stereoscopic image.^[15]^ Stereopsis is acquired after birth and built on the basis of simultaneous perception and binocular fusion. Having stereopsis involves having a good understanding of 3-dimensional space. People who have stereopsis can perform delicate operations. Stereopsis is the real advantage of binocular vision.^[16]^

Development of binocular vision in children occurs from 2 months to 9 years, and the developmental peak of binocular vision occurs when children are 1 to 3 years-old.^[17]^ The sensitive period of binocular vision development occurs before children are 3 years-old, and the mature stage of binocular vision development occurs when children are 4 to 9 years-old. Development of binocular vision requires the 2 eyes to have similar vision. During the entire binocular vision development period, any abnormal visual experience, especially the anomalous retinal correspondence caused by a nonparallel visual axis, forces the development of binocular vision to be suspended.^[18]^ A large number of studies have shown that a surgical operation can effectively correct residual strabismus, which can lead the binocular visual axis to become parallel, stabilize the eye position, and establish normal retinal correspondence as well as promote the recovery of binocular visual function and establish far and near stereopsis. The effects caused by adverse factors during this sensitive period of visual development are reversible. If we remove these adverse factors in time, binocular vision can develop continuously and patients will still have a chance to form good stereopsis. Therefore, strabismus should be treated as soon as it is discovered. For the recovery of binocular vision after surgery in children, if the treatment occurs after the visual sensitivity period, the curative effect will be less than treatment during this visual sensitivity period.^[19]^ Recent clinical studies have shown that some of the children who receive treatment after 9 years-old also recover their binocular vision. This research tells us that surgery is still beneficial for the recovery of binocular vision in children during the whole developmental period of vision and binocular vision still has the opportunity to develop.^[20]^ For older children, we should also conduct the operation and compliment it with binocular vision training afterwards. We should not give up helping patients obtain good binocular vision. Nevertheless, the choice of operation time is critical for children.

According to the literature, some scholars believe that the nonaccommodative component that cannot be corrected by wearing full hyperopic correction glasses should be operated as soon as possible and because it is very important for the recovery of binocular vision.^[9]^ One report stated that some patients had medial rectus fibrosis and their abduction was limited due to the long absence of surgical treatment after the onset of strabismus. Therefore, it is bad for children to wait to be treated for a long time after the onset of strabismus. Other scholars believe that children with partially accommodative esotropia should be fully observed and that we should treat the nonaccommodative component after the strabismus angle is stable.^[21]^ In this study, we try to find an appropriate preoperative conservative treatment time and to study the effects of the preoperative wearing time on postoperative outcomes to provide useful guidance for clinical treatment.

Our research data showed that half a year after the operation, 13 patients who wore glasses for half a year before the operation regained far stereopsis and 11 patients regained near stereopsis. Among patients who wore glasses for 1 year before the operation, 16 patients regained far stereopsis and 15 patients regained near stereopsis. There was no statistically significant difference between the 2 groups, which showed that the 2 groups had similar outcomes. Therefore, for children with partially accommodative esotropia, conducting the operation when wearing full hyperopic correction glasses for half a year can correct eye position and improve binocular vision as early as possible. If surgery cannot be performed after the patient has worn glasses for half a year due to various subjective and objective factors, a suitable outcome can still be obtained if surgery is performed after the patient has worn glasses for 1 year.

Operations for all children were carried out according to the residual esotropia that could not be corrected by wearing glasses. The strabismus angle that bilateral medial rectus recession can correct is + 50△, so we adopted bilateral medial rectus recession combined with unilateral external rectus shortening for children whose strabismus angle was more than + 50△. The technique depends not only on the strabismus angle but also on the width and intensity of the muscle as well as the location of the muscle insertion. These procedures require the surgeon to have abundant experience.^[22]^

We checked the strabismus angle with glasses in children after surgery. For children who fulfill the diagnostic criteria of overcorrection (>-8△), we should reduce the degree of the hyperopia glasses appropriately. For children who fulfill the diagnostic criteria for undercorrection (> + 8△), if the postoperative strabismus angle is more than + 15△, a second operation is required to correct the angle. The postoperative strabismus angle of children in our study was less than + 15△, so we overcorrected the glasses of these children according to the postoperative results after cycloplegia and asked them to come in for regular check-ups.^[3]^ With the development of vision, physiological hyperopia decreases year after year and the eye position can change after surgery, which might require us to use 1% atropine eye gel to achieve cycloplegia for finding change of the children's refractive diopter every half year, so that we can maintain the normal eye position and obtain the best corrected vision by refitting them with appropriate lenses. Children who have stable eye positions but still have amblyopia should continue to receive treatment for amblyopia.^[7]^ Binocular vision is a satisfactory standard for evaluating the treatment effectiveness of strabismus and amblyopia. For children with partially accommodative esotropia, correction of the eye position and improvement of appearance is not our final goal. Instead, the recovery of binocular vision is our ultimate goal. For all children, postoperative functional training should be carried out to promote the establishment of binocular vision and allow children to obtain a better visual experience. We should tell the parents of children preoperatively that the operation can only correct the nonaccommodative component and that the children still need to correct the residual esotropia with glasses after the operation.

## Acknowledgment

Special thanks to professor Qi Zhao for the guidance of the article and the guidance of professor Qigui Liu on the statistical method of the article.

## Author contributions

**Conceptualization:** Jiahuan Zhang.

**Data curation:** Dong Han.

**Formal analysis:** Danni Jiang.

**Funding acquisition:** Danni Jiang.

**Investigation:** Danni Jiang.

**Methodology:** Danni Jiang.

**Project administration:** Danni Jiang.

**Resources:** Danni Jiang.

**Software:** Tianxu Pei.

**Supervision:** Danni Jiang.

**Validation:** Danni Jiang.

**Visualization:** Danni Jiang.

**Writing – original draft:** Danni Jiang.

**Writing – review & editing:** Qi Zhao.
